# Anastomotic Biliary Stricture Development after Liver Transplantation in the Setting of Retained Prophylactic Intraductal Pediatric Feeding Tube: Case and Review

**DOI:** 10.1155/2018/4707389

**Published:** 2018-09-30

**Authors:** Patrick T. Koo, Valentina Medici, James H. Tabibian

**Affiliations:** ^1^Division of Gastroenterology and Hepatology, Department of Internal Medicine, University of California Davis Medical Center, Sacramento, California, USA; ^2^Division of Gastroenterology, Department of Medicine, Olive View-UCLA Medical Center, Sylmar, California, USA

## Abstract

The biliary anastomosis remains a common site of postoperative complications in liver transplantation (LT). Biliary complications have indeed been termed the “Achilles' heel” of LT, and while their prevention, diagnosis, and treatment have continued to evolve over the last two decades, various challenges and uncertainties persist. Here we present the case of a 33-year-old man who, 10 years after undergoing LT for idiopathic recurrent intrahepatic cholestasis, was noted to have developed pruritus and abnormalities in serum liver biochemistries during routine post-liver transplant follow-up. Abdominal ultrasound revealed a linear, 1.5 mm hyperechoic filling defect in the common bile duct; magnetic resonance cholangiopancreatography demonstrated a curvilinear filling defect at the level of the choledochocholedochostomy, corresponding to the ultrasound finding, as well as an anastomotic biliary stricture (ABS). On endoscopic retrograde cholangiography (ERC), a black tubular stricture with overlying sludge was encountered and extracted from the common bile duct, consistent with a retained 5 Fr pediatric feeding tube originally placed at the time of LT. The patient experienced symptomatic and biochemical relief and successfully underwent serial ERCs with balloon dilatation and maximal biliary stenting for ABS management. With this case, we emphasize the importance of ensuring spontaneous passage or removal of intraductal prostheses placed prophylactically at the time of LT in order to minimize the risk of chronic biliary inflammation and associated sequelae, including cholangitis and ABS formation. We also provide herein a brief review of the use of prophylactic internal transanastomotic prostheses, including biliary tubes and stents, during LT.

## 1. Introduction

The biliary anastomosis, typically constructed via choledochocholedochostomy, remains the most common anatomical site for postoperative complications in LT. Indeed, biliary complications have been regarded as the “Achilles' heel” of LT and are a source of significant morbidity. We present a case of late anastomotic biliary stricture (ABS) development presenting 10 years after LT for idiopathic recurrent intrahepatic cholestasis in the setting of an intraductally retained 5 Fr pediatric feeding tube placed prophylactically at the time of LT and provide a synopsis of the rationale for, use, and outcomes of prophylactic transanastomotic prostheses, including biliary tubes and stents, during LT.

## 2. Case Report

The patient is a 33-year-old man with a history of progressive idiopathic recurrent intrahepatic cholestasis diagnosed initially at the age of 15. He was managed with ursodeoxycholic acid, cholestyramine, rifampin and naloxone but eventually failed medical therapy as evidence by the development of cirrhosis complicated by ascites, esophageal variceal hemorrhage, pruritus, and progressively rising Model for End-Stage Liver Disease score.

At the age of 22, the patient underwent deceased-donor LT. At the time of LT, the patient's native bile duct was noted to be 10 mm in diameter, while the donor bile duct was 2.5mm in diameter. In order to make the two orifices more congruent, a ductoplasty was performed with part of the recipient bile duct being oversewn and an end-to-end choledochocholedochostomy being created with running circumferential 5-0 absorbable sutures. The total cold ischemia time was 8 hours, 32 minutes, and the total warm ischemia time was 41 minutes. There were no intraoperative complications, and the patient recovered well following surgery. He was managed on tacrolimus and did well for a decade. Approximately half-way through this period, for geographical and insurance reasons, the patient transferred LT care to our institution. At his 10 year post-LT appointment, the patient endorsed new-onset generalized pruritus and was noted to have developed multiple albeit relatively minor abnormalities in his serum liver test profile, with an alkaline phosphatase of 121 (normal: 35-115 U/L), aspartate aminotransferase of 53 U/L, alanine aminotransferase of 68 U/L, and total bilirubin of 1.2 mg/dL.

**Figure 1:**
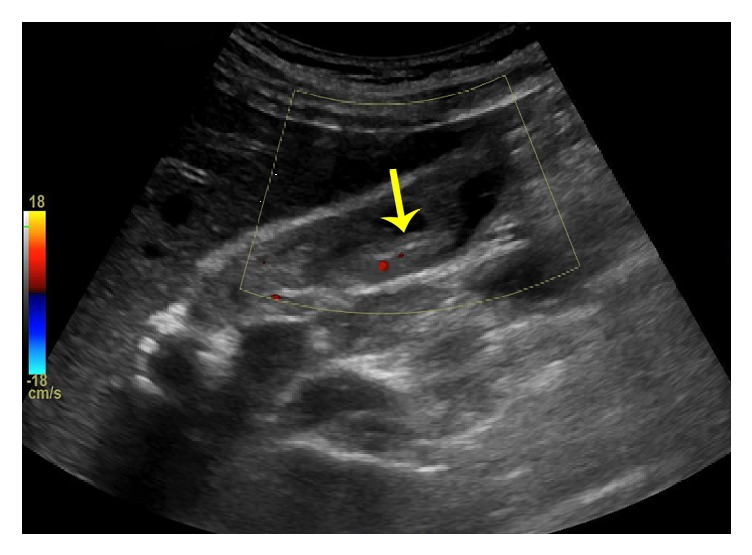
Abdominal ultrasound with filling defect in bile duct.

**Figure 2:**
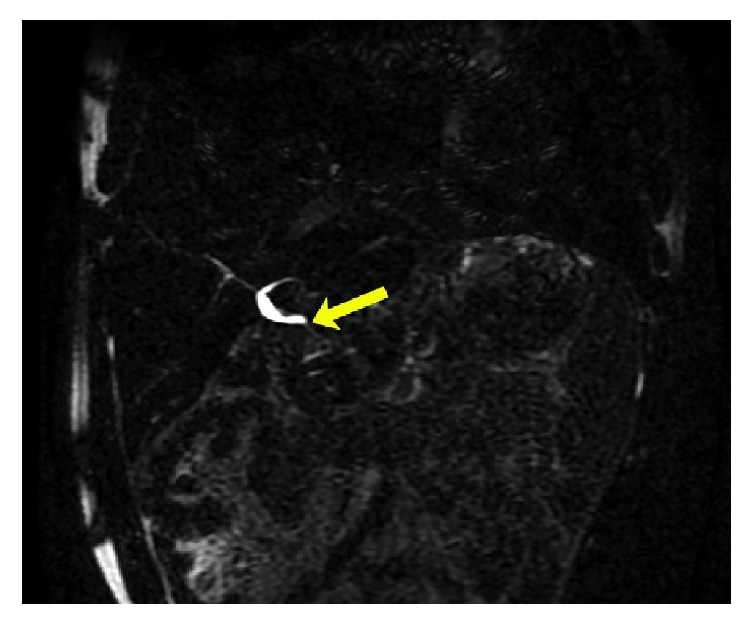
MRI/MRCP showing anastomotic biliary stricture, common hepatic ductal dilatation, and subtle curvilinear filling defect within the common hepatic duct.

Abdominal ultrasound showed no evidence of intrahepatic or extrahepatic biliary ductal dilatation but did note a linear filling defect within the common bile duct (CBD) as seen in Figure 1. MRI/MRCP was thus performed, which was significant for an abrupt change in caliber at the biliary anastomosis consistent with stricture (ABS), dilatation of the common hepatic duct (CHD) to 7 mm, and a curvilinear filling defect at the level of the anastomosis (Figure 2). Given these findings, the patient's original LT operative report was retrieved from the performing institution; this revealed that in addition to the ductoplasty, a 3.5cm segment of 5 Fr pediatric feeding tube had been placed through the biliary anastomosis to serve as a temporary internal stent.

**Figure 3:**
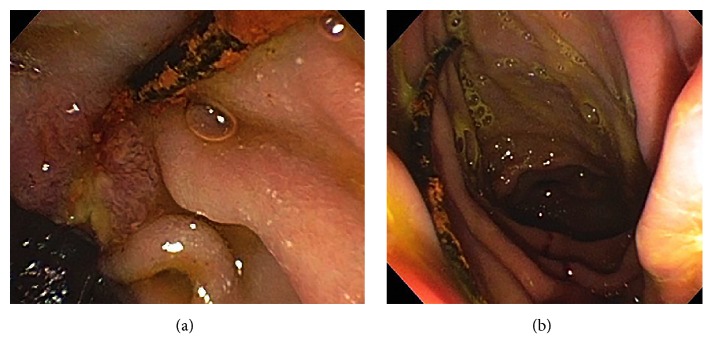
(a) ERC showing black pediatric feeding tube with overlying biofilm protruding from the papilla. (b) ERC with successful extraction of black pediatric feeding tube from the papilla.

**Figure 4:**
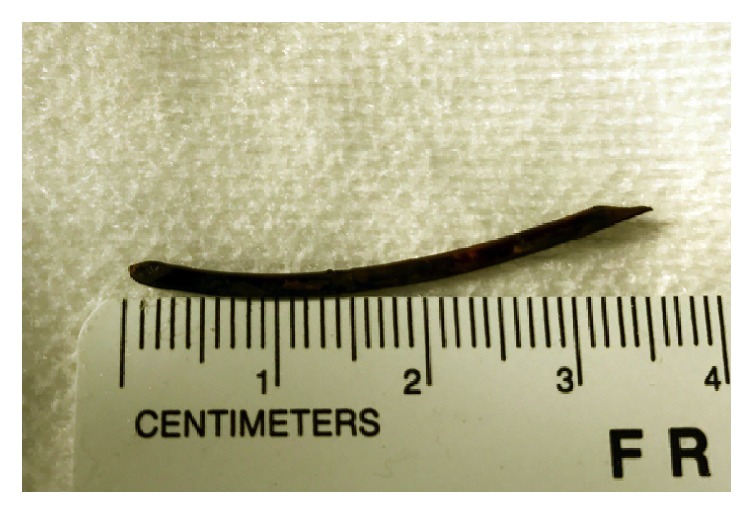
Extracted pediatric feeding tube.

Given the patient's new-onset pruritus, worsening serum liver tests, and abnormal imaging findings, endoscopic retrograde cholangiography (ERC) was performed. This revealed a small, tortuous native (i.e., recipient) CBD and a short ABS with proximal CHD dilatation. The ABS was first balloon dilated to 6 mm followed by sweeping of the extrahepatic duct was performed with a 9 mm extraction balloon; this first yielded biliary sludge, but with additional sweeps, a 3.5 cm long, black tubular structure with overlying sludge/biofilm was extracted (Figures 3(a) and 3(b)). This was grasped and brought out* per os* using forceps and appeared to be consistent with an oxidized pediatric feeding tube (Figure 4). An 8 mm × 4 cm CRE balloon was then used to further dilate the ABS, and two 10 Fr x 7 cm Cotton-Leung plastic biliary stents were deployed across it. In doing so, the patient's serum laboratory tests returned to baseline, and his pruritus resolved over the ensuing weeks. The patient now nears completion of serial ERCs at three month intervals as part of a 1-year long maximal stenting protocol in order to achieve durable ABS resolution [1].

## 3. Discussion

The biliary anastomosis represents the most common anatomical site for postoperative complications in LT and is a cause of significant morbidity. Accordingly, biliary complications have been termed the “Achilles' heel” of LT [2–4]. Biliary strictures are among the most frequent major complications involving the anastomosis and can be classified as either anastomotic (ABS) or nonanastomotic based on location, appearance, and suspected etiology [5]. With respect to the former, a recent meta-analysis of over 14,000 LTs found the incidence of ABS to be approximately 13% [2]. ABSs can develop at any time following LT, but the majority present within one year of LT (mean 5-8 months) [2]. ABSs in the early postoperative period are usually related to surgical technique and/or mismatch of recipient and donor bile ducts, while late-onset ABSs are believed to be secondary to fibrosis from preceding local ischemia or chronic inflammation-related injury [5, 6]. Historically, several surgical techniques have existed for constructing the biliary anastomosis, with additional measures being employed to deal with significant size mismatch between the donor and recipient bile ducts, as in the case of our patient [7, 8]. Early biliary reconstructions included loop choledochojejunostomy, Roux-en-Y choledochojejunostomy (RYCJ), and using the gallbladder as a conduit [7, 8]. In the 1980s, duct-to-duct anastomosis became the most popular technique. Compared to RYCJ and similar surgical techniques, the duct-to-duct anastomosis has the theoretical advantage of no bowel manipulation, less biliary reflux by virtue of an intact sphincter, potentially decreased cholangiocarcinoma risk, and easier endoscopic access (when needed for biliary intervention) [7, 8]. For a number of years, the duct-to-duct anastomosis was constructed over a percutaneous T-tube (irrespective of bile duct size discrepancy) in an effort to bridge the biliary anastomosis and thereby decrease the risk of ABS formation [9]. However, T-tubes have been associated, at least in some studies, with higher rates of biliary complications, especially leakage from the T- tube exit site, and over the last couple decades are thus no longer routinely used in LT at most centers [2, 10]. In cases where there is significant size mismatch between the donor and recipient bile ducts, additional measures which have been employed include: partially closing a patulous recipient CHD or CBD, spatulating the donor duct, everting the recipient CBD, creating a common orifice between the cystic and common ducts, side-to-side ductal anastomosis, or choledochoduodenostomy. These techniques may be performed with or without the use of temporary internal stents.

The use of an internal stent across the biliary anastomosis theoretically eliminates the possible complications associated with T-tubes while preserving the integrity of the biliary anastomosis. Johnson et al. were the first to review the use of prophylactic internal transanastomotic biliary stenting at the time of LT [11]. They placed 6Fr double-J ureteral stents in a transanastomotic, transpapillary fashion and reported a significantly lower biliary complication rate in patients with internal stenting compared to those with T-tubes (18% versus 38%) [11]; there was also a trend towards a lower incidence of ABS development in the internal stent group (7.6% versus 11%) [11].

Multiple studies have since followed with somewhat conflicting findings and thus a lack of convincingly beneficial results. For example, Trachart et al. studied the use of 8 Fr x 2 cm internal stents fabricated from a T-tube and reported a lower rate of ABS compared to the nonstent group (5% versus 15.1%) [12]. Similarly, Jung et al., using 6-8 Fr silastic stents, reported a statistically significant lower ABS rate in the stent group compared to the nonstent group (3.23% versus 11.76%) [13]. In contrast, Mathur et al. found that the use of transanastomotic, transpapillary 5-8Fr silastic pediatric feeding tubes were associated with higher rates of ABS, although the difference was not statistically significant [14]. Mathur et al. concluded that prophylactic internal stenting did not reduce risk of biliary complications and was instead associated with a higher adjusted risk for requiring endoscopic interventions (e.g., ERC) within the first 90 days post-LT [14].

In an effort to minimize the need for endoscopic procedures for stent removal, studies have since been performed using synthetic bioabsorbable stents [15]. Janousek et al. investigated the use of transanastomotic bioabsorbable biliary stents, made from polydioxanone monofilaments. None of the patients in the stent group developed biliary strictures; however, the sample size was small, and long term follow-up was not reported [15]. Most recently, the first randomized control trial assessing the efficacy of prophylactic transanastomotic stenting for duct-to-duct biliary reconstruction in LT was performed [16]. Santosh Kumar et al. randomized LT recipients to either receive a transanastomotic, transpapillary 3-5 Fr ureteric stent or no stent; the authors reported a trend towards higher stricture formation in the stent group compared to the nonstent group (22.6% versus 6.1%), although this was not statistically significant [15]. Given a significantly higher risk of bile leak in the stent group, the trial was terminated early [16].

While the aforementioned studies have varied in the type and size of stents used, the fashion in which they were placed, and the duration for which they were left* in situ*, it is important to note that they all ensured stent passage or removal (or bioabsorption). Johnson et al. reported that 43% of internal stents passed spontaneously, while the remaining 57% required endoscopic removal [11]. Similarly, Tranchart et al. reported that 95% of their patients with internal stents required endoscopic removal, as only 5% of stents were noted to have passed spontaneously [12]. Therefore, it is imperative that sight not be lost of these stents, and that appropriate follow-up (and if needed, removal) be performed, as with other pancreatobiliary stents.

In our patient, while mismatch in donor and recipient bile duct size was a potential risk factor for the development of ABS, it is likely that the prolonged presence of a foreign object in the form of a retained transanastomotic pediatric feeding tube served as a nidus of chronic, low-grade inflammation and ultimately contributed to the development of the patient's late-onset ABS. Therefore, stent removal was recommended, and in doing so, the patient benefitted from symptomatic, biochemical, and cholangiographic improvement.

While we await a more definitive verdict on the efficacy of temporary prophylactic transanastomotic biliary stents in LT, it is clear that if placed, non-bioabsorbable intraductal stents should be removed (if spontaneous passage is not observed) to minimize the risk of developing complications down the road, including cholangitis and/or ABS formation. Our case emphasizes the importance of the routine use of imaging as well as other safeguards following intraductal prosthesis placement to either document spontaneous stent passage or identify patients who may require further evaluation and potentially endoscopic stent removal.

## Abbreviations

ABS: Anastomotic biliary stricture
ERC: Endoscopic retrograde cholangiography
LT: Liver transplantation.
